# Complete rupture of the lateral rectus muscle following pterional craniotomy

**DOI:** 10.12701/jyms.2026.43.46

**Published:** 2026-07-09

**Authors:** Chan-Ho Cho, Won Jae Kim

**Affiliations:** Department of Ophthalmology, Yeungnam University College of Medicine, Daegu, Korea

A 71-year-old woman presented with binocular horizontal diplopia that developed immediately after aneurysm clipping via left pterional craniotomy. She had hypertension that was controlled with medication. Visual acuity was 20/25 in the right eye and 20/50 in the left eye. An ocular motility examination demonstrated 45 prism diopters (PD) esotropia in the primary position, with complete loss of abduction in the left eye (Fig. 1). Orbital magnetic resonance imaging (MRI) was performed to evaluate extraocular muscle integrity; however, the evaluation was not definitive. The patient was followed up for 6 months, during which no spontaneous improvement in ocular motility was observed. Therefore, strabismus surgery was planned. Intraoperative exploration revealed a complete rupture of the left lateral rectus muscle (LR) (Fig. 2). The distal muscle stump was identified and noted to be retracted; however, the proximal end could not be located despite thorough dissection, confirming total muscle rupture. A 6-mm recession of the ipsilateral medial rectus muscle was performed, combined with temporal transposition of the superior rectus muscle. Six months postoperatively, the patient showed residual 12 PD esotropia and 6 PD left hypertropia in the primary position, with subjective improvement in diplopia.

Pterional craniotomy is a commonly used neurosurgical approach for the management of anterior circulation aneurysms [1]. Although this technique provides excellent exposure of the frontal and temporal lobes, it can occasionally lead to ophthalmological complications because of its proximity to the orbit. Reported complications include ptosis, diplopia, and restrictive strabismus, which may result from mechanical tethering or adhesion of the extraocular muscles to the surgical defect [2]. To the best of our knowledge, complete extraocular muscle rupture, specifically of the LR, has not been previously reported. Patel et al. investigated the incidence and clinical significance of frontal sinus entry, orbital entry, and both during pterional craniotomy for microsurgical aneurysm clipping [3]. They reported that frontal sinus or orbital entry was common after pterional craniotomy. Violation of the orbital wall, particularly the superolateral orbital rim, occurs more frequently than frontal sinus entry. However, it rarely leads to clinically significant ocular complications. Our case illustrates that when an orbital violation occurs, direct injury to the extraocular muscle, including complete LR rupture, is possible. Meticulous protection of the periorbita, gentle and well-irrigated drilling, and minimal manipulation of the orbital roof may be necessary to minimize the risk of extraocular muscle damage [1,2].

Although the contour and continuity of the extraocular muscles are generally assessable on orbital imaging, LR rupture was not definitively identified on preoperative MRI in this case. A previous report on extraocular muscle injury also noted that imaging studies may not always fully delineate the extent or location of muscle damage [4]. In the present case, this limitation may have been related to orbital soft tissue changes following the preceding pterional craniotomy, slice thickness or partial-volume effects, marked retraction of the muscle stump, and the presence of thin fibrous tissue obscuring the true discontinuity. Given the rarity of complete extraocular muscle rupture after pterional craniotomy, direct muscle injury may not be initially suspected. Thus, severe and persistent motility limitations should prompt careful clinical correlation and consideration of surgical exploration, even when imaging findings are inconclusive.

Early recognition of unintended extraocular muscle injury is crucial. In patients who develop new-onset diplopia and marked limitation of extraocular motility after pterional craniotomy, particularly when orbital wall violation has occurred, muscle rupture or severe mechanical compromise should be promptly suspected rather than attributing the symptoms solely to transient neuropathy or postoperative edema. In such cases, early ophthalmologic consultation and close collaboration between neurosurgeons and strabismus specialists are critical for prompt diagnostic workup and appropriate surgical planning.

## Figures and Tables

**Fig. 1.**
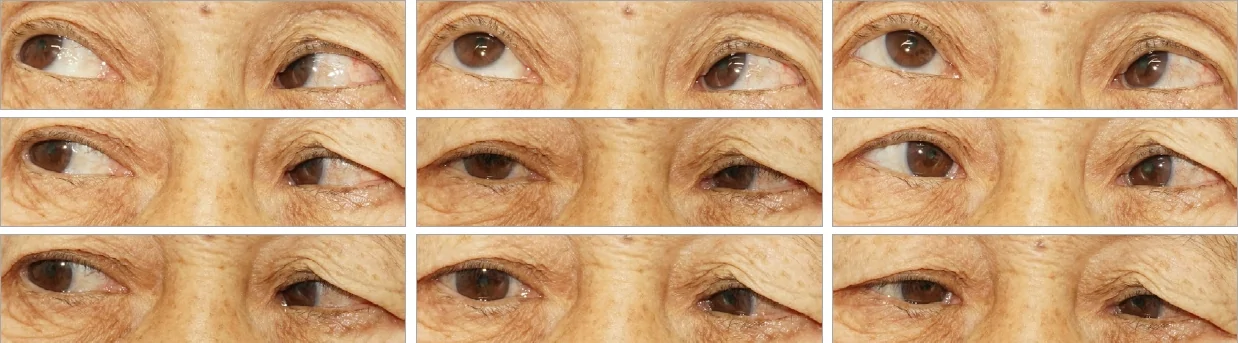
Ocular motility examination at the initial visit showing the nine diagnostic positions of gaze. Note the marked esotropia of the left eye in primary position and complete loss of abduction.

**Fig. 2.**
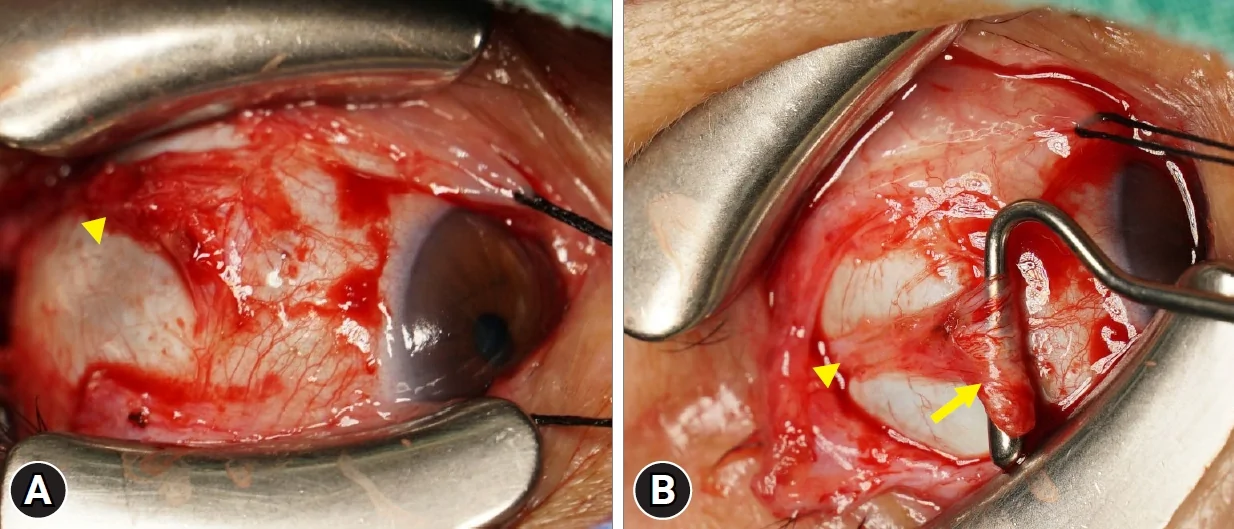
Intraoperative findings of the left lateral rectus muscle. (A) Thin fibrous tissue remnant along the presumed course of the lateral rectus muscle (arrowhead). (B) Retracted distal stump of the lateral rectus muscle (arrow) and adjacent fibrous tissue remnant (arrowhead). The proximal stump could not be identified despite careful posterior dissection, consistent with complete rupture of the lateral rectus muscle.
